# A Rare Case of an Isolated Left Adrenal Haematoma in Blunt Force Trauma

**DOI:** 10.7759/cureus.27131

**Published:** 2022-07-21

**Authors:** Sara Izwan, William Anderson

**Affiliations:** 1 General Surgery, Robina Hospital, Gold Coast, AUS; 2 School of Medicine and Dentistry, Griffith University, Gold Coast, AUS

**Keywords:** accidental trauma, unilateral adrenal haemorrhage, low impact injury, adrenal haemorrhage, left adrenal gland

## Abstract

Isolated left adrenal gland injuries following blunt abdominal trauma are extremely rare, accounting for only 1.5-4% of all adrenal trauma cases. Most traumatic injuries are right-sided and associated with other concurrent organ injuries. While acute, unilateral adrenal injuries can be asymptomatic, it is important to recognise the potentially life-threatening complications from haemorrhage and/or adrenal insufficiency. Due to its rarity, there are currently no established guidelines for management, monitoring, or follow-up of adrenal gland trauma. We present a case report of a rare, isolated, post-traumatic left adrenal gland haemorrhage. A 39-year-old man presented with mild abdominal pain following a mountain bike accident. Initial assessment revealed tenderness in the left upper quadrant with normal vital signs and biochemistry, but computer tomography (CT) imaging demonstrated an enlarged left adrenal gland with regional fat stranding and free fluid consistent with an acute adrenal haematoma. He was admitted to the ward for observation and discharged on day three of admission. At a six-week follow-up, he reported a complete resolution in symptoms. Follow-up CT of the adrenals at five months post-injury demonstrated complete resolution of the isolated adrenal haematoma. Post-traumatic isolated left adrenal gland injuries are rare in the reported literature, and this case highlights the lack of current guidelines for management and monitoring in cases of isolated adrenal haemorrhage. The current consensus appears to be appropriate with conservative management and follow-up with serial abdominal CT until the resolution of haemorrhage and/or symptoms.

## Introduction

Isolated trauma to the adrenal gland is a rare and underreported injury, with an incidence of between 1.5% and 4% of all adrenal trauma cases [1-4]. Due to its unique anatomy and the mechanism required to result in an isolated injury, adrenal injury is typically unilateral and right-sided [2,5-8]. Adrenal gland injury is rare due to its small size, deep retroperitoneal position, and presence of fatty tissue surrounding it; however, the adrenal glands are vulnerable to bleeding due to their extensive network of collateral circulation [9].

In the setting of trauma, adrenal injury can point to other concomitant injuries and is associated with increased morbidity and mortality. Because unilateral adrenal injury is typically a benign condition, management is generally conservative with analgesia and observation, whereas surgery is usually indicated for associated injury to other internal organs, including the spleen, kidneys, bowel, liver, or ribs. Although mostly self-limiting, acute adrenal injury can be life-threatening if it represents a significant cause of bleeding or hypotension, or results in bilateral injury which increases the risk of adrenal insufficiency or adrenal crisis.

Contrast-enhanced computed tomography (CT) of the abdomen remains the gold standard in distinguishing adrenal haematomas from incidental adenomas in the setting of trauma [6,9,10]. It is important for the clinician to be aware that acute adrenal haemorrhage could be secondary to a traumatic insult, or from a pre-existing adrenal lesion. However, given its rarity, there is a lack of established guidelines for monitoring and follow-up of isolated adrenal trauma in the literature. We present this case to raise awareness of this uncommon injury and the risks of associated lethal adrenal crises, as well as present a recommendation for conservative management, observation, and follow-up of patients at six weeks and three months post-injury.

## Case presentation

**Figure 1:**
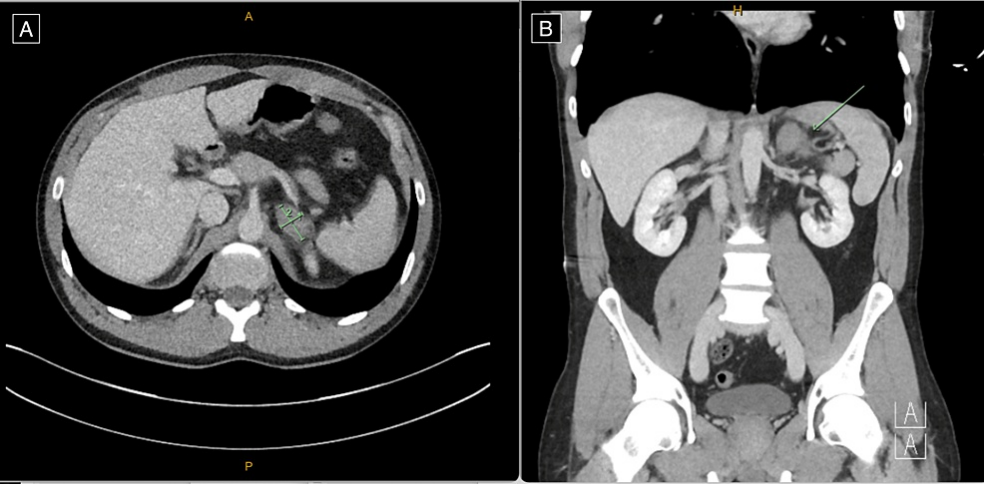
Computed tomography of the abdomen and pelvis demonstrating enlargement of the left adrenal gland with associated regional fat stranding and trace regional free fluid, in keeping with a traumatic adrenal gland haematoma, measuring 32 × 20 mm (A) on axial view and (B) on coronal view (see arrows).

We present the case of a 39-year-old male who presented to the emergency department with left upper quadrant pain following an injury to the abdomen from a medium-speed mountain bike accident. The mechanism of injury was a fall coming off a jump, where he landed on the handlebars of his bike. He had no concurrent medical or previous surgical history and was not on any anticoagulants. Initial assessment revealed minimal left upper quadrant abdominal pain and normal vital signs on presentation without evidence of hypotension (blood pressure of 136/93 mmHg) or tachycardia (heart rate of 78 beats per minute, regular in rhythm). Urinalysis was negative for haematuria. CT imaging of the abdomen revealed a 32 × 20 mm enlarged left adrenal gland with regional periadrenal fat stranding and free fluid consistent with an isolated left-sided adrenal haemorrhage (Figure 1).

**Figure 2:**
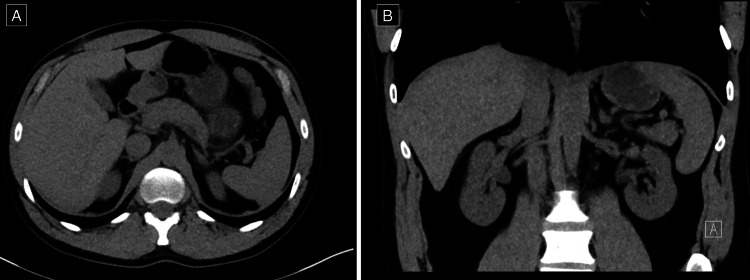
Computed tomography adrenals demonstrating complete resolution of the left adrenal haematoma on (A) axial view and (B) coronal view at five months follow-up.

No other injuries were identified on CT. Haemoglobin on presentation was 163 g/L. He was admitted to the ward under the General Surgery team for observation and remained haemodynamically stable throughout hospital admission. Haemoglobin, renal function, and biochemical markers remained within normal limits. He was discharged on day three of admission. At six weeks follow-up, he reported feeling very well with no abdominal pain, was passing normal bowel motions, and no further concerns were raised. Follow-up CT adrenals at five months post-injury demonstrated complete resolution of the isolated adrenal haematoma (Figure 2).

## Discussion

The adrenals are small retroperitoneal glands directly below the diaphragm and are attached to the crura of the diaphragm by the renal fascia. The left adrenal is crescent in shape and surrounded by the stomach and spleen. The right adrenal gland is more likely to be injured given its anatomical position close to the spinal column and drainage through the right adrenal vein through the inferior vena cava, whereas the left adrenal vein joins the left renal vein. As a result, the right adrenal vein is more susceptible to contusions and oedema, and therefore the right adrenal gland is more likely to undergo acute injury. This appears to be consistent with many reports in the international literature [7] and is similar in incidence to a large retrospective analysis performed recently by an Australian trauma institute [8].

Case reports of adrenal injuries are not new but are important to acknowledge given their association with injuries to surrounding structures and risks of haemodynamic instability. At the time of this report, there has been only one other case report published of an isolated left adrenal gland injury from blunt trauma [11], with one case published in 2020 describing an isolated injury to the left adrenal gland sustained from a penetrating gunshot wound [12]. The most recent review of the literature with regards to an isolated unilateral adrenal injury is a paper by Lehrberg and Kharbutli that also recommends follow-up with serial abdominal CTs until resolution of both symptoms and on radiological imaging [1].

Isolated adrenal gland injuries with no other organ involvement are usually self-limiting, without the need for major operative intervention. The most common symptom is abdominal pain, and it does not produce any specific symptoms or derangement in biochemical markers. In most cases, unilateral adrenal haemorrhage may be asymptomatic, but bilateral adrenal haemorrhage can cause a primary adrenal insufficiency, which is a potentially life-threatening disorder [13]. Clinicians must also consider the possibility of an underlying pre-existing adrenal neoplasm as a cause of haemorrhage [14].

CT imaging is the gold standard for detecting adrenal gland injury as in all trauma cases. Common findings include hyperdensity of the gland indicative of a haematoma, periadrenal fat standing, oval or round lesions, and ipsilateral diaphragmatic crural thickening [8]. A 50-90 Houndsfield Unit (HU) hyperdense adrenal lesion associated with periadrenal fat stranding should be considered an adrenal haemorrhage in unenhanced CT images, especially if there is a history of trauma [15]. Unfortunately, no HU was reported in our case. Magnetic resonance imaging (MRI) remains the most sensitive and specific method for diagnosing adrenal haemorrhage but is not indicated in the acute trauma setting.

Surgery (exploratory laparotomy, adrenalectomy) and interventional radiologic procedures (embolisation) may be indicated in haemodynamically unstable trauma patients. For patients with accompanying visceral haemorrhage, angiography has been postulated as an alternative treatment option to laparotomy [16]; however, surgery is still indicated in cases of uncontrolled bleeding. Most adrenal gland injuries are treated conservatively [14,17]. In the literature, current guidelines suggest that management of adrenal injuries depends on the extent of the injury, the status of the contralateral adrenal gland, haemodynamics of the patient, and presence (or absence) or associated injuries [1,16]. Conservative management is increasingly recommended for blunt adrenal gland trauma and has yielded good prognoses for most patients. Resolution in uncomplicated, acute adrenal haematomas has been suggested in two to four weeks [1,17].

## Conclusions

There is no consensus or established guidelines for monitoring and follow-up of isolated adrenal haemorrhage. There is a need for dedicated classification and/or grading of adrenal injuries to guide the clinician in terms of management and prognosis. Current recommendations include follow-up with serial abdominal CTs until the resolution of haemorrhage and symptoms, and a high degree of suspicion for adrenal insufficiency in cases of unexplained hypotension in unilateral or bilateral adrenal gland injury. Some studies have also suggested MRI as an alternative imaging method for evaluating adrenal haemorrhage, as bleeding can be seen in different signals in the acute, subacute, and chronic stages. In our case, we recommend for uncomplicated acute isolated adrenal injuries in haemodynamically stable patients, appropriate management includes admission for analgesia and observation, with follow-up at four to six weeks in the outpatient setting, and consideration of repeat abdominal CT imaging to assess progress at that time.
